# The clinical, biochemical and genetic features associated with *RMND1*-related mitochondrial disease

**DOI:** 10.1136/jmedgenet-2016-103910

**Published:** 2016-07-13

**Authors:** Yi Shiau Ng, Charlotte L Alston, Daria Diodato, Andrew A Morris, Nicole Ulrick, Stanislav Kmoch, Josef Houštěk, Diego Martinelli, Alireza Haghighi, Mehnaz Atiq, Montserrat Anton Gamero, Elena Garcia-Martinez, Hana Kratochvílová, Saikat Santra, Ruth M Brown, Garry K Brown, Nicola Ragge, Ahmad Monavari, Karen Pysden, Kirstine Ravn, Jillian P Casey, Arif Khan, Anupam Chakrapani, Grace Vassallo, Cas Simons, Karl McKeever, Siobhan O'Sullivan, Anne-Marie Childs, Elsebet Østergaard, Adeline Vanderver, Amy Goldstein, Julie Vogt, Robert W Taylor, Robert McFarland

**Affiliations:** 1 Wellcome Trust Centre for Mitochondrial Research, Institute of Neuroscience, Newcastle University, Newcastle upon Tyne, UK; 2 Neuromuscular and Neurodegenerative Disease Unit, Children Research Hospital Bambino Gesù, Rome, Italy; 3 Department of Genetic Medicine, Central Manchester University Hospitals NHS Foundation Trust, St Mary's Hospital, Manchester, UK; 4 Department of Neurology, George Washington University Medical School, Children's National Health System, Washington, DC, USA; 5 First Faculty of Medicine, Institute for Inherited Metabolic Disorders, Charles University in Prague, Prague, Czech Republic; 6 Institute of Physiology, Academy of Sciences of the Czech Republic, Prague, Czech Republic; 7 Division of Metabolism, Children Research Hospital Bambino Gesù, Rome, Italy; 8 Department of Genetics, Harvard Medical School, Boston, Massachusetts, USA; 9 Department of Medicine and the Howard Hughes Medical Institute, Brigham and Women's Hospital, Boston, Massachusetts, USA; 10 Department of Pediatrics, Aga Khan University, Karachi, Pakistan; 11 Pediatric Nephrology Unit, Hospital Universitario Reina Sofia, Cordoba, Spain; 12 Department of Pediatrics and Adolescent Medicine, First Faculty of Medicine, Charles University in Prague and General University Hospital in Prague, Prague, Czech Republic; 13 Department of Clinical Inherited Metabolic Disorders, Birmingham Children's Hospital NHS Foundation Trust, Birmingham, UK; 14 Oxford Medical Genetics Laboratories, Oxford University Hospitals NHS Foundation Trust, The Churchill Hospital, Oxford, UK; 15 Clinical Genetics Unit, West Midlands Regional Genetics Service, Birmingham Women's NHS Foundation Trust, Birmingham, UK; 16 National Centre for Inherited Metabolic Disorders, Temple Street Children's University Hospital, Dublin, Ireland; 17 Department of Paediatric Medicine, Leeds General Infirmary, Leeds, UK; 18 Department of Clinical Genetics, Copenhagen University Hospital Rigshospitalet, Copenhagen, Denmark; 19 Department of Clinical Genetics, Temple Street Children's University Hospital, Dublin, Ireland; 20 Leicester Children's Hospital, Leicester Royal Infirmary, Leicester, UK; 21 Department of Metabolic Medicine, Great Ormond Street Hospital NHS Foundation Trust, London, UK; 22 Department of Paediatric Neurology, Central Manchester University Hospitals NHS Foundation Trust, Manchester, UK; 23 Institute for Molecular Bioscience, University of Queensland, St. Lucia, Queensland, Australia; 24 Department of Paediatric Medicine, The Royal Belfast Hospital for Sick Children, Belfast, UK; 25 Division of Child Neurology, Children's Hospital of Pittsburgh, Pittsburgh, Pennsylvania, USA; 26 Department of Medical and Molecular Genetics, Centre for Rare Diseases and Personalised Medicine, School of Clinical and Experimental Medicine, University of Birmingham, Birmingham, UK; 27 Faculty of Health and Life Sciences, Oxford Brookes University, Oxford, UK

**Keywords:** mitochondrial respiratory chain deficiencies, renal disease, congenital sensorineural deafness, prognosis, lactic acidosis

## Abstract

**Background:**

Mutations in the *RMND1* (Required for Meiotic Nuclear Division protein 1) gene have recently been linked to infantile onset mitochondrial disease characterised by multiple mitochondrial respiratory chain defects.

**Methods:**

We summarised the clinical, biochemical and molecular genetic investigation of an international cohort of affected individuals with *RMND1* mutations. In addition, we reviewed all the previously published cases to determine the genotype–phenotype correlates and performed survival analysis to identify prognostic factors.

**Results:**

We identified 14 new cases from 11 pedigrees that harbour recessive *RMND1* mutations, including 6 novel variants: c.533C>A, p.(Thr178Lys); c.565C>T, p.(Gln189*); c.631G>A, p.(Val211Met); c.1303C>T, p.(Leu435Phe); c.830+1G>A and c.1317+1G>T. Together with all previously published cases (n=32), we show that congenital sensorineural deafness, hypotonia, developmental delay and lactic acidaemia are common clinical manifestations with disease onset under 2 years. Renal involvement is more prevalent than seizures (66% vs 44%). In addition, median survival time was longer in patients with renal involvement compared with those without renal disease (6 years vs 8 months, p=0.009). The neurological phenotype also appears milder in patients with renal involvement.

**Conclusions:**

The clinical phenotypes and prognosis associated with *RMND1* mutations are more heterogeneous than that were initially described. Regular monitoring of kidney function is imperative in the clinical practice in light of nephropathy being present in over 60% of cases. Furthermore, renal replacement therapy should be considered particularly in those patients with mild neurological manifestation as shown in our study that four recipients of kidney transplant demonstrate good clinical outcome to date.

## Introduction

Mitochondrial disease is clinically and genetically heterogeneous and often causes multisystem manifestations. Defects in mitochondrial protein synthesis secondary to mutations in mitochondrial tRNA synthetases, mitochondrial ribosomal proteins and mitochondrial elongation factors are increasingly recognised and identified through next-generation sequencing.^1^ Mutations in the *RMND1* (Required for Meiotic Nuclear Division protein 1) gene cause multiple mitochondrial respiratory chain deficiencies and were first linked to human disease in 2012.^2^ ^3^ Recent findings suggest that *RMND1* plays an important role in mitochondrial translation by anchoring or stabilising the mitochondrial ribosome near the site of mRNA maturation.^3^ ^4^

The clinical phenotypes associated with *RMND1* mutations are expanding, ranging from a fatal, infantile encephalomyopathy with lactic acidosis^2^ ^5^ to a less severe phenotype characterised by developmental delay, congenital sensorineural deafness, hypotonia and renal disease.^4^ ^6^ In this study, we identified new patients harbouring recessive mutations in *RMND1* from several metabolic clinics and research centres across Europe (UK, Ireland, Italy, Denmark, Spain and Czech Republic) and the USA. We aimed to describe the phenotype–genotype correlate and determine the prognostic factors in survival by combining all other previously reported cases.

## Subjects and methods

### Subjects

Clinical and laboratory data were collated using a standardised data collection form. We also conducted a literature review to ascertain previously published cases, approaching respective authors for additional data wherever required. This study was performed in accordance with the World Medical Association’s Declaration of Helsinki, research and ethical guidelines issued by each of our institutions.

### Mitochondrial histochemistry

For patients who underwent muscle biopsy, oriented muscle blocks were subjected to cytochrome *c* oxidase (COX), succinate dehydrogenase (SDH) and sequential COX/SDH histochemical reaction to evaluate the numbers of COX-deficient fibres as a marker of mitochondrial respiratory chain deficiency.^7^ The SDH reaction was also used to determine the number of ‘ragged-blue’ fibres, whereby such muscle fibres exhibit increased levels of this enzyme activity in the subsarcolemmal region. The same histochemical studies were applied to study the cardiac and kidney tissues.

### Identification of pathogenic *RMND1* mutations

The selection criteria of patients with suspected mitochondrial disease for whole exome sequencing (WES) and the interpretation of results have been described elsewhere.^6^ ^8^ ^9^ Sanger sequencing was applied to verify the *RMND1* mutations and study segregation within the pedigree. Where the *RMND1* mutation(s) were identified by candidate gene sequencing, the coding region (11 coding exons) and intron–exon boundaries of the *RMND1* gene were amplified using M13-tagged primers, and the resultant Sanger sequencing chromatograms were compared with the *RMND1* reference sequence (GenBank Accession Number NM_017909.2). Ensembl was used to investigate amino acid conservation of novel *RMND1* variants.

### Statistical analysis

Kaplan-Meier analysis and Cox-regression analysis were applied to determine the survival and associated prognostic factors. All analyses were performed using SPSS software (V.22.0) and significance level (p value) was determined at ≤0.05 level.

## Results

### Clinical phenotypes

The clinical features of 32 patients (male:female=12:20) from 21 pedigrees are summarised in table 1. Fourteen patients from 11 families (P1–P9, P10.1, P10.2, P11.1) are new cases; their clinical details are provided in online supplementary data 1. Authors from the previously published cases^5^ ^6^ ^8^ (P11.2, P12–P18.2) also completed the case report form and provided additional data for this study (P11.2, P12–P16, online supplementary data 1). Clinical data were extracted from the literature for the remaining eight patients (P19 to P21.5).^2–4^ ^10^ The frequencies of individual clinical features are outlined in table 2.

**Table 1:** Summary of all cases (n=32)

Patient	Ethnicity	Onset/current age (year)	SNHL	DD	FTT	MC	Sz	Tone	Renal	HTN	Cardiac	↑ Lact	MRC deficiency (muscle)	RMND1 mutation (cDNA/aa change)
1 (F)	Italian	At birth/4	+	+	+	+	+	C	CD, RTA, ESRF, A	+	−	+	CIV	c.713A>G p.(Asn238Ser); c.1303C>T p.(Leu435Phe)
2 (M)	Caucasian	0.17/10.4	+	+	−	n.s.	−	C	ESRF	+	HCM	+	CIV	c.713A>G p.(Asn238Ser); c.565C>T p.(Gln189*)
3 (F)	Irish	At birth/*3.1	+	+	+	+	−	Normal	RTA, ESRF	+	HCM, PDA, PT	+	CI, CIV	c.713A>G p.(Asn238Ser); c.533C>A p.(Thr178Lys)
4.1 (M)	Caucasian	0.5/8	+	+	−	−	+	C, P	−	−	−	−	n.d.	Homozygous c.713A>G p.(Asn238Ser)
4.2 (M)	Caucasian	At birth/6	+	+	−	−	+	C, P	−	−	−	−	n.d.	Homozygous c.713A>G p.(Asn238Ser)
5 (F)	Caucasian, native American	At birth/9	+	+	+	+	+	C, P	CD, ESRF, Tx	+	−	+	n.d.	Homozygous c.713A>G p.(Asn238Ser)
6 (M)	European, Mexican	At birth/7.67	+	+	+	−	−	C	CD, RTA, ESRF, Tx	+	Mild LVH	+	CI, CIII, CIV	c.713A>G p.(Asn238Ser); c.1317+1G>T, p.?
7.1 (F)	Caucasian	1/11	+	+	−	−	−	Normal	CKD stage 2, A	−	−	MA	n.d.	c.713A>G p.(Asn238Ser); c.1250G>A p.(Arg417Gln)
7.2 (F)	Caucasian	1/8	+	+	−	−	−	Normal	CKD stage 3, A	−	−	MA	n.d.	c.713A>G p.(Asn238Ser); c.1250G>A p.(Arg417Gln)
8 (F)	Caucasian	At birth/3.75	+	+	+	+	+	Normal	RTA, CKD stage 4	+	Normal	+	CIV	c.631G>A p.(Val211Met); c.830+1G>A p.(Met244Glyfs*20)
9 (M)	Pakistan	0.11/*6	+	+	+	+	+	C	RTA, CKD	+	HB	+	n.d.	Homozygous c.1349G>C p.(*450Serext*31)
10.1 (F)	Bangladesh	0.3/*0.94	+	+	+	−	−	C	RTA, ESRF, A	+	Pericardial effusion, HB	+	CI, CIV	Homozygous c.1349G>C p.(*450Serext*31)
10.2 (F)	Bangladesh	0.75/*3	+	+	+.	−	−	C	RTA, ESRF, A	+	HB and had PPM	+	n.d.	Homozygous c.1349G>C p.(*450Serext*31)
11.1 (F)	Pakistan	0.67/*1.33	+	+	+	+	−	C	CD (autopsy)	−	HB	+	n.d.	Homozygous c.1349G>C p.(*450Serext*31)
11.2 (F)	Pakistan	0.25/*1	+	+	+	+	−	C	RTA, A	−	Normal	+	CI, CIV	Homozygous c.1349G>C p.(*450Serext*31)
12 (F)	Pakistan	1.5/*6.67	+	+	+	+	−	C	CD, RTA, ESRF	n.s.	DCM	−	CI, CIII, CIV	Homozygous c.1349G>C p.(*450Serext*31)
13 (F)	Pakistan	0.5/*0.53	+	+	+	+	−	C	RTA	+	Small VSD, HB	+	CI, CIII, CIV	Homozygous c.1349G>C p.(*450Serext*31)
14 (M)	Pakistan	0.5/*5.8	+	+	+	−	−	C	CD, RTA, ESRF	+	HB	+	CI, CIII, CIV	Homozygous c.1349G>C p.(*450Serext*31)
15 (F)	Pakistan	0.08/*2	+	+	+	+	−	C	CD, ESRF	n.s.	HB	−	CI, CIII, CIV	Homozygous c.1349G>C p.(*450Serext*31)
16 (F)	Irish	0.11/*3.4	+	+	+	+	+	P	RTA, CKD stage 4	+	−	+	CI, CIV	c.713A>G p.(Asn238Ser); c.829_830+2het_delGAGT p.?
17.1 (M)	Sudanese	At birth/*0.92	−	+	n.s.	+	+	C,P	−	−	−	+	CI, CIII, CIV	Homozygous c.1250G>A p.(Arg417Gln)
17.2 (M)	Sudanese	At birth/*4 days	−	n.s.	n.s	n.s.	+	C, P	−	−	−	+	n.d.	Homozygous c.1250G>A p.(Arg417Gln)
18.1 (F)	Caucasian	1.17/17	+	+	+	−	−	C	Proteinuria, ESRF, Tx	+	−	n.d.	CI, CIII, CIV	c.713A>G p.(Asn238Ser); c.1003delG p.(Ala335Leufs*2)
18.2 (F)	Caucasian	At birth/14	+	+	+	−	+	C	Proteinuria, ESRF, A, Tx	+	−	n.d.	CI, CIII, CIV	c.713A>G p.(Asn238Ser); c.1003delG p.(Ala335Leufs*2)
19 (M)	Caucasian	At birth/*4.25	+	+	+	−	+	C	RTA, A, CD (autopsy)	+	LVH	+	CI, CIV	c.613G>T p.(Asp205Cysfs*4); c.713A>G p.(Asn238Ser)
20.1 (F)	n.s.	0.17/*1.08	n.s.	n.s.	n.s.	+	+	C	−	n.s.	n.d.	+	n.d.	Homozygous c.1250G>A p.(Arg417Gln)
20.2 (F)	n.s.	Day 6/*0.42	n.s.	n.s.	+	+	+	C	−	n.s.	n.d.	+	Low CIV in fibroblast	Homozygous c.1250G>A p.(Arg417Gln)
21.1 (M)	Saudi Arabian	At birth/*1.5	n.s.	n.s.	n.s.	n.s.	+	C	−	n.s.	n.d.	+	CIV	Homozygous c.504+1G>A, p.?
21.2 (M)	Saudi Arabian	At birth/*12 days	n.s.	n.s.	n.s.	n.s.	n.s.	C	−	n.s.	n.d.	+	n.d.	Homozygous c.504+1G>A, p.?
21.3 (F)	Saudi Arabian	At birth/*8 months	n.s.	n.s.	n.s.	n.s.	n.s.	C	−	n.s.	n.d.	+	n.d.	Homozygous c.504+1G>A, p.?
21.4 (M)	Saudi Arabian	At birth/*4 months	n.s.	n.s.	n.s.	n.s.	n.s.	C	−	n.s.	n.d.	+	n.d.	Homozygous c.504+1G>A, p.?
21.5 (F)	Saudi Arabian	Stillborn	n.s.	n.s.	n.s.	n.s.	n.s.	n.d.	−	n.s.	n.d.	n.d.	n.d.	Homozygous c.504+1G>A, p.?

*, Deceased, ↑, Lact, raised serum lactate; A, anaemia; C, central hypotonia; CI, complex I; CIII, complex III; CIV, complex IV; CD, cystic dysplasia; CKD, chronic kidney disease; DCM, dilated cardiomyopathy; DD, developmental delay; ESRF, end stage renal failure; F, female; FTT, failure to thrive; HB, heart block; HCM, hypertrophic cardiomyopathy; HTN, hypertension; LA, lactic acidosis; LVH, left ventricular hypertrophy; M, male; MA, metabolic acidosis with normal serum lactate; MC, microcephaly; MRC, mitochondrial respiratory chain; n.d., no data; n.s., not stated; P, peripheral spasticity; PDA, patent ductus arteriosus; PPM, permanent pacemaker; PT, pulmonary hypertension; RTA, renal tubular acidosis/persistent hyponatraemia and hyperkalaemia; SNHL, sensorineural hearing loss; Sz, seizure; Tx, renal transplant; VSD, ventricular septal defect.

**Table 2:** Frequency of clinical features associated with *RMND1* mutations (n=32)

Clinical features	Percentage		
	Present	Absent	Not stated
Neurological and developmental			
Hypotonia	75	16	9
Sensorineural hearing loss	72	6	22
Developmental delay	75	–	25
Seizure	44	44	12
Failure to thrive	53	19	28
Microcephaly	41	34	25
Peripheral spasticity	19	56	25
Lactic acidaemia	62	19	19
Renal	66	34	–
Gastrointestinal	47	25	28
Dysmorphic appearance/ congenital deformity	41	28	31
Hypertension	47	25	28
Cardiac	38	41	21

10.1136/jmedgenet-2016-103910.supp1 Supplementary data

#### Antenatal and birth history

Oligohydramnios was identified in five pregnancies and polyhydramnios was detected in one pregnancy. Intrauterine growth retardation affected two pregnancies. All but three pregnancies were full term (≥37 weeks) except P1, P3 and P20.2; the earliest delivery (P3) was at 31 weeks gestation. Eight patients required respiratory resuscitation at birth. One patient was stillborn at 34 weeks gestation (P21.5).

#### Neurological

The most common clinical features associated with *RMND1* mutations were hypotonia (n=24, 75%) and global developmental delay (n=24, 75%) followed by sensorineural hearing loss (n=23, 72%) that was most frequently identified at neonatal hearing screening. Other common neurological features that prompted medical referral and investigations were failure to thrive (n=17, 53%), seizures (n=14, 44%), microcephaly (n=13, 41%) and peripheral spasticity associated with central hypotonia (n=6, 19%). Strabismus was detected in four patients (P5, P6, P8 and P16) and two underwent corrective surgery.

**Figure 1:**
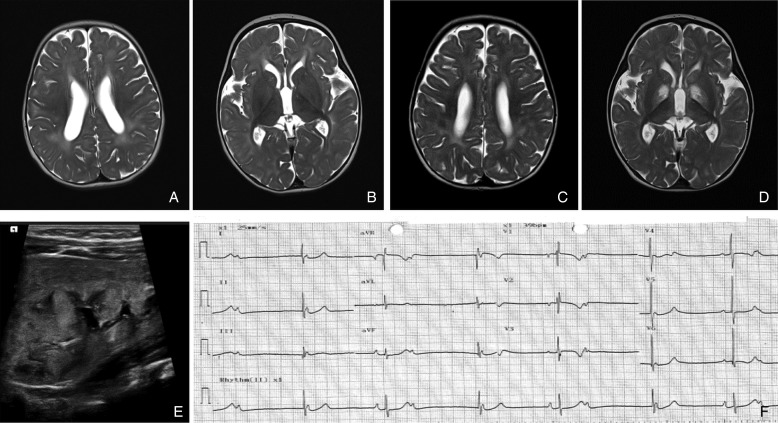
Radiological imaging. (A–D) Axial T2-weighted MRI head from P1. (A) There were prominent T2 hyperintensities in the white matter suggestive of delayed myelination and (B) basal ganglia appeared normal at 6 months. Repeat imaging (C) showed improvement of the white-matter abnormality but there were new, symmetrical changes in the basal ganglia (D) at 2 years old. (E) Renal ultrasound showed dysplastic kidney in P1. (F) Twelve-lead ECG of P10.2 showed atrioventricular (AV) dissociation and bradycardia (heart rate 39 bpm).

Brain imaging was available for analysis in 22 patients, revealing abnormalities in 17 patients. White matter abnormalities were identified in 14 patients, of whom four of them had additional cystic changes in the cerebral lobe(s) (figure 1A–D). Basal ganglia calcification was identified on CT head in three patients. Acute ischaemic infarct involving the unilateral parietal–temporal area was identified in one patient (P12). No brainstem abnormalities were observed and five patients had a normal cranial MRI.

EEG was available for review in 10 patients. The EEG changes were non-specific with a variable degree of background slowing and low amplitude being the most common findings (n=6) and epileptic discharge was captured in four patients. Three patients had febrile seizures (P4.1, P4.2 and P18.2) of whom only one developed on-going epilepsy (generalised seizure and myoclonus, P18.2). Infantile spasm was reported in two patients (P1 and P8) though typical pattern of hypsarrhythmia was not present in one of them (P8).

#### Metabolic derangement and renal involvement

Lactic acidaemia (2.2–29 mmol/L, normal range <2.2 mmol/L) was documented in 20 patients (63%). CSF lactate level was measured in seven patients and was generally lower (1.5–5.9 mmol/L) than the serum lactate level except in one patient. Renal involvement was evident in 21 patients (66%). The manifestations of kidney disease included different stages of chronic kidney disease (CKD, n=17), arterial hypertension (n=15), persistent hyponatraemia and hyperkalaemia (which were suggestive of renal tubular acidosis type 4, n=13), dysplastic or hypoplastic kidneys (n=8, figure 1E) and normocytic anaemia (n=9). Hypouricosuric hyperuricaemia was identified in three patients (P1, P7.1 and P7.2). Two patients had metabolic acidosis without evidence of lactic acidaemia (P7.1 and P7.2). Urinary electrolyte levels were available in three cases, which showed high urinary sodium and low potassium levels. Results of short-synacthen test were available in two patients and were normal. Tubulointerstitial changes were identified in three renal biopsies.

Twelve patients developed end-stage renal failure (ESRF), which was fatal for seven of them. Four patients (P5, P6, P18.1 and P18.2) have been treated with dialysis followed by renal transplant.

#### Gastrointestinal

Fifteen patients were established on enteral feeding for failure to thrive and/or dysphagia. Two patients had recurrent pancreatitis with a history of diarrhoea and abdominal pain (P1 and P19). Abdominal ultrasound showed hyperechogenic pancreas in two patients (P1 and P7.2). A patient who was born prematurely developed necrotising enterocolitis required laparotomy and bowel resection (P3).

#### Cardiac

Hypertrophic cardiomyopathy/left ventricular hypertrophy was identified in four patients (P2, P3, P6 and P19) and one patient had dilated cardiomyopathy (P12). Congenital cardiac defects including small ventricular septal defect (n=1) and patent ductus arteriosus with pulmonary hypertension (n=1) were also identified. Of the nine patients with a homozygous c.1349G>C, p.(*450Serext*32) mutation, clinically significant bradycardia (variable degrees of heart block, figure 1F) was present in seven cases, of whom two required emergency cardiac pacing (P10.1 and P10.2).

#### Other findings

Dysmorphic features or congenital abnormalities were identified in 13 patients: bilateral equinus foot deformity (n=4), hypertrichosis (n=2), anteriorly rotated ears, tent mouth and rocker bottom feet (n=1), inverted nipples and stellate irises (n=1), developmental dysplastic hip (n=1), large anterior fontanelle, small toes and small suboptimally curved pinna (n=1) and a non-specific dysmorphic appearance (n=3). Five patients had skin changes including hypopigmented lesions (n=2), pale and doughy skin (n=1), pigmented skin rash in trunk and dry, thickened skin (n=1), as well as intermittent cutis marmorata suggestive of dysautonomia (n=1). Two siblings with hypopigmented lesions also had pili torti (P7.1 and P7.2).

### Findings of muscle biopsies, fibroblast studies and other tissues

**Figure 2:**
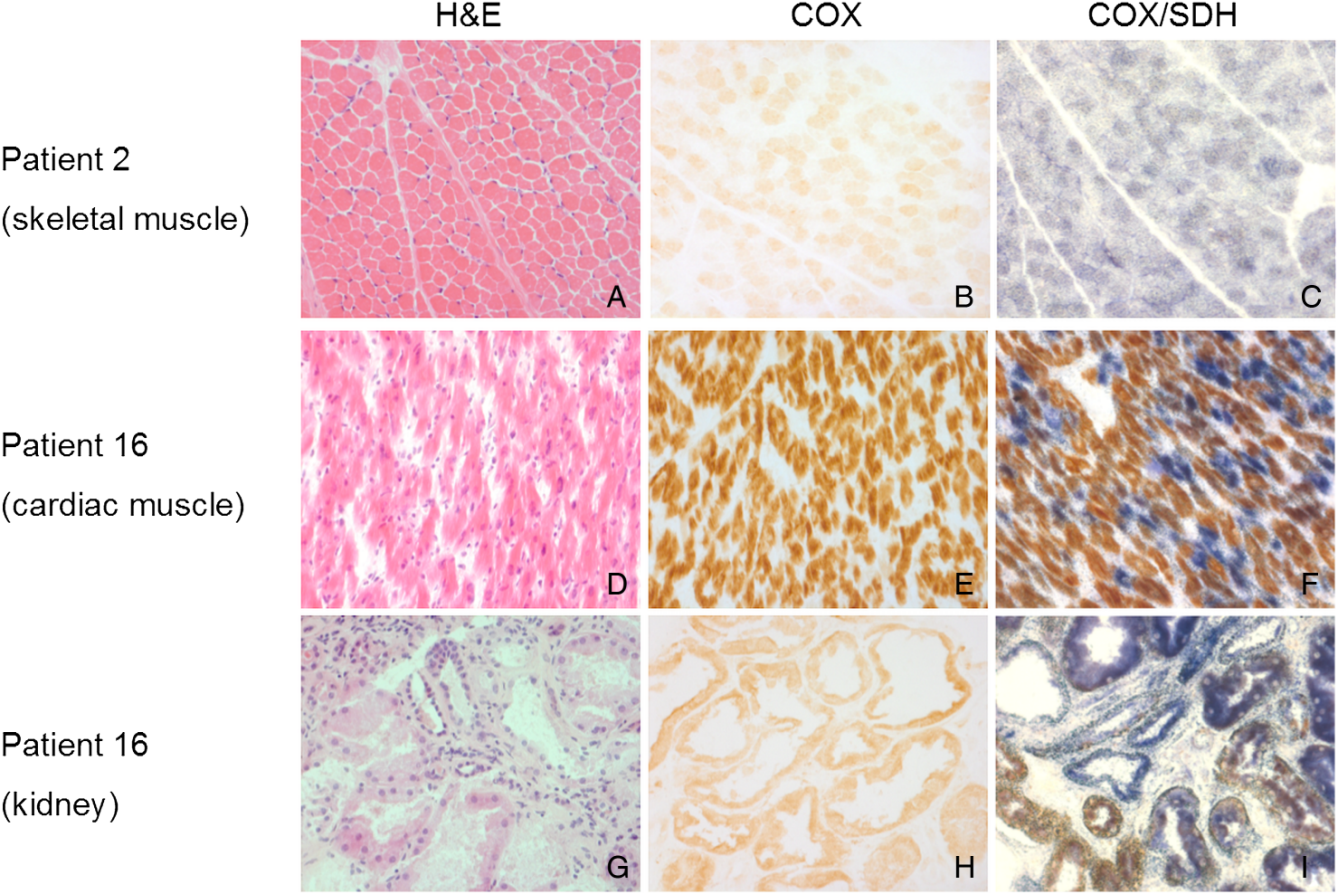
Histopathological and histochemical studies. (A–C) Skeletal muscle biopsy from P2; (D–F) postmortem cardiac tissue from P16; (G–I) postmortem kidney tissue from P16. Marked *c* oxidase (COX)-deficient muscle fibres (c) and renal tubules (i), lesser extent of COX deficiency in cardiomyocytes was identified through sequential COX/succinate dehydrogenase (SDH) histochemical reaction.

Histopathological and histochemical description of muscle biopsies was available in 11 patients: variation in fibre size (n=4/6), type I fibre grouping (n=3/6), increased lipid content (n=3/6), ragged-red fibres (n=5/10) and COX-deficient fibres (n=8/9) (figure 2A). None of the muscle biopsies showed inflammatory changes. Measurement of mitochondrial (mt) DNA copy number was performed in eight patients and it showed either normal (n=6) or increased (n=2) mtDNA copy number.

Biochemical studies of muscle biopsy material were performed in 17 patients; 7 had combined complex I, III and IV deficiencies, 5 had complex I and IV deficiencies and 5 had an isolated CIV deficiency.

Respiratory chain function was evaluated in cultured fibroblasts of nine patients. Normal respiratory chain activities were identified in three patients, isolated complex IV deficiency was present in five patients and only one patient had multiple respiratory chain deficiencies.

Histochemical studies of postmortem cardiac and renal tissues from P16 (figure 2D–I) revealed extensive COX deficiency.

### Pathogenic variants in *RMND1* gene

**Figure 3:**
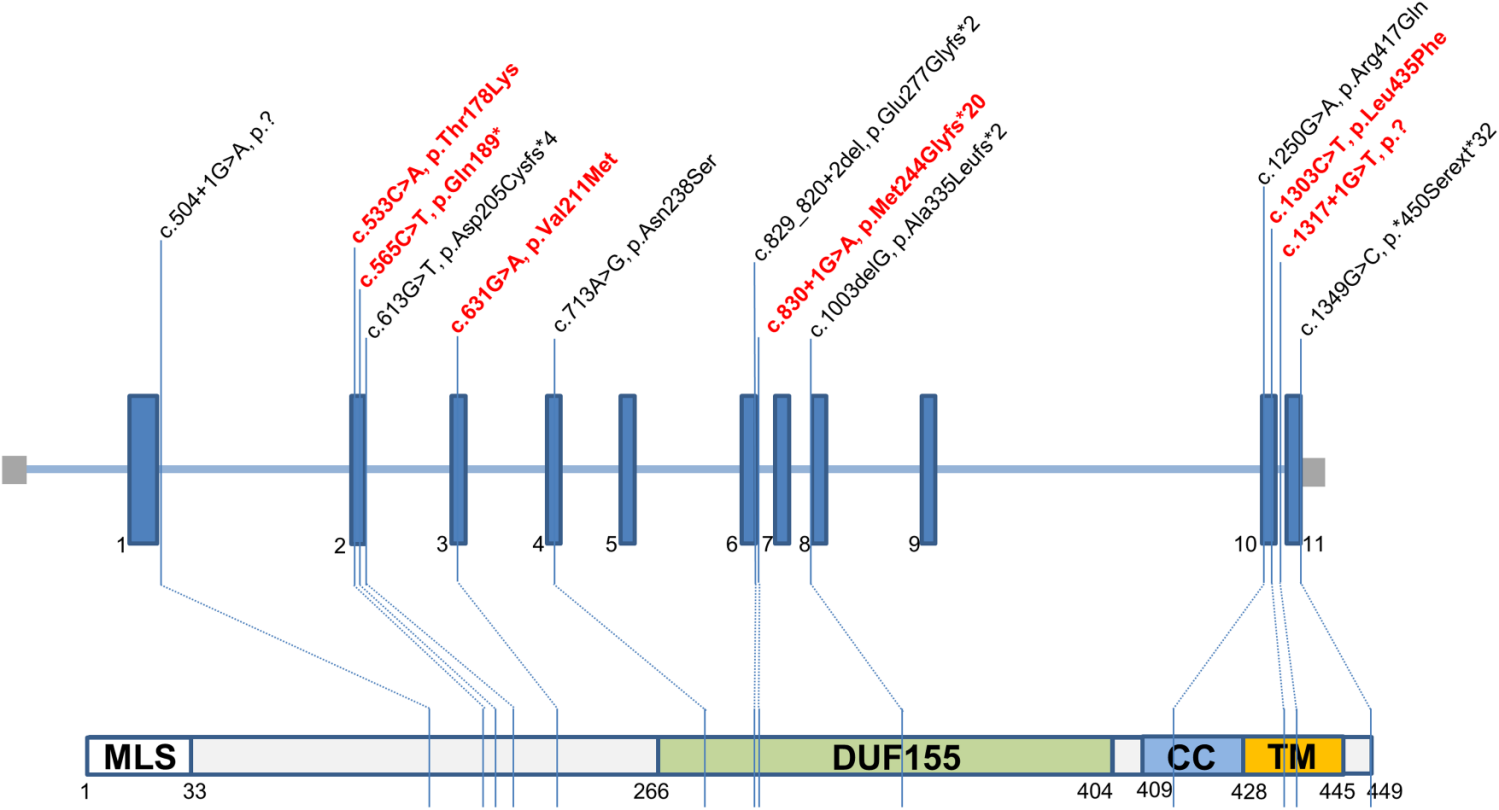
Pathogenic variants in *RMND1* gene (n=13). Six novel variants are depicted in red font.

Thirteen pathogenic variants were identified of which seven had been reported previously c.1349G>C, p.(*450Serext*32*); c.713A>G, p.(Asn238Ser); c.829_830+2het_delGAGT, p.?; c.1250G>A, p.(Arg417Gln); c.504+1G>A (aberrant splicing), c.1003delG, p.(Ala335Leufs*2) and the remaining six were novel: c.533C>A, p.(Thr178Lys); c.565C>T, p.(Gln189*); c.631G>A, p.(Val211Met), c.1303C>T, p.(Leu435Phe); c.830+1G>A and c.1317+1G>T splicing variants (figure 3). The p.Thr178, p.Val 211 and p.Leu435 variants are highly conserved, supportive of pathogenicity. Three *RMND1* mutations (p.(*450Serext*32), p.(Asn238Ser) and p.(Arg417Gln)) were identified in multiple families; the remaining nine mutations were unique to individual families. The missense mutation c.1349G>C, p.(*450Serext*32) was exclusively found in South Asian ethnicities (seven Pakistani and one Bangladesh families), while the c.713A>G, p.(Asn238Ser) variant was identified in 10 Caucasian families. All novel *RMND1* variants have been submitted to ClinVar (submission ID numbers: SCV000258932—SCV000258940).

### Associated factors for survival

**Figure 4:**
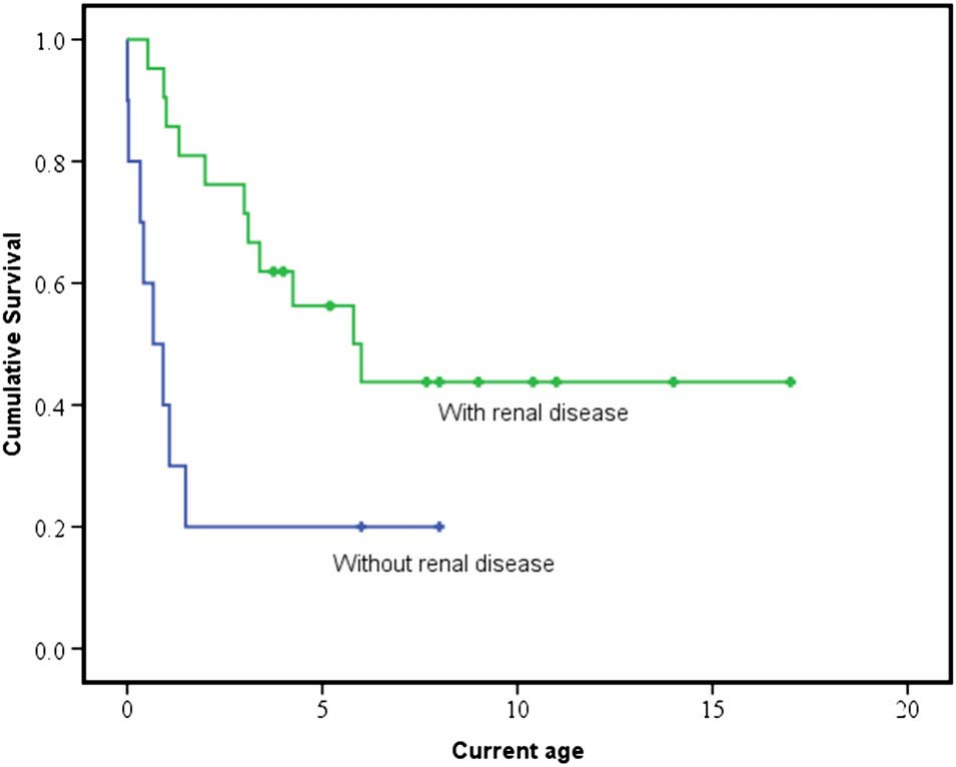
Kaplan-Meier curves comparing survival between patients with and without renal involvement. Censored data (represent the number of patients that are still alive at their most recent clinical review) are shown as crosses. The median survival time in patients with renal involvement (green) is significantly longer than those without renal involvement (blue), 6.0 years versus 8 months (log-rank test, p=0.009).

The median age of disease onset was 29 days (Q1=at birth, Q3=0.5 year, range at birth to 1.5 years, P21.5 excluded from analysis) and all of them presented before 2 years old; 20 patients were deceased with nine of these aged under 1 year. The median survival time was 6.0 years for patients with renal involvement (95% CI 2.8 to 9.2 years) but only 8 months for those without renal disease (95% CI at birth to 1.4 years) (log-rank test, p=0.009), as illustrated in figure 4.

The presence of renal disease (p=0.017) and later disease onset (p=0.028) were associated with a longer survival (n=27, five cases excluded due to incomplete data) using Cox regression multivariate analysis (median duration of follow-up was 3.4 years, range: 0.01–17.0 years). Seizure (p=0.066), hypotonia (p=0.996) and gender (p=0.102) were not associated with survival.

## Discussion

*RMND1* encodes a protein composed of 449 amino acids that is targeted to the inner mitochondrial membrane.^2^ It belongs to the evolutionarily conserved sif2 family of proteins that share the DUF155 domain.^3^ Recent studies have suggested that RMND1 acts to anchor or stabilise the mitochondrial ribosome near the sites of mRNA maturation, spatially coupling post-transcriptional handling of mRNAs with their translation.^3^ ^4^ Recessive mutations in *RMND1* result in a generalised mitochondrial translation defect and multiple mitochondrial respiratory chain deficiencies.

The main findings of our study are as follows: (1) hypotonia, developmental delay and congenital sensorineural deafness are cardinal clinical features of this disease; (2) there is a continuum of clinical phenotype and severity associated with *RMND1* mutations, ranging from, at the most severe end, infantile encephalomyopathy with early death to childhood-onset nephropathy associated with longer survival; the oldest patient is currently 17 years; (3) while renal disease progressed to ESRF in 12 patients, kidney transplant appears to be helpful, with four patients remaining well without significant progression of their existing neurological deficit following transplantation; (4) bradycardia has been observed only in patients who harbour a homozygous c.1349G>C, p.(*450Serext*32) variant, which likely represents a South Asian founder mutation (eight families); (5) the c.713A>G, p.(Asn238Ser) variant has only been identified in Caucasian families (n=10), to date; (6) multiple respiratory chain deficiencies were the most frequently identified biochemical abnormality in muscle, although uncommon in patient fibroblasts, with abnormal respiratory chain activities being observed in only one of eight patients.

Global neurodevelopmental delay affects more than two-thirds of the patients. The delay in gross motor development frequently occurs with the presence of hypotonia, which is most likely mediated centrally. This is supported by the identification of white-matter abnormalities in MR imaging,^4^ which is suggestive of delayed myelination. In addition, five patients with initial hypotonia subsequently developed peripheral hypertonia and spasticity. Some of these clinical pictures and radiological findings are similar to the congenitally acquired TORCH (Toxoplasmosis, Other (Syphilis), Rubella, Cytomegalovirus and Herpes Simplex Virus) infection,^11^ but this can be rapidly excluded with serological testing. The language developmental delay is, in part, confounded by severe sensorineural deafness and correction with hearing aids or cochlear implants results in some improvement. The degree of learning disability is variable among the older patients, ranging from those with mild-to-moderate disability and attending special school (P2, P6, P7.1, P7.2, P18.1 and P18.2) to verbalisation of only a few words at age 9 (P5).

There are a number of clinical and biochemical features of *RMND1* mutations, for example, congenital sensorineural deafness, lactic acidaemia, hypotonia and multiple mitochondrial respiratory chain deficiencies, which are also described in other genetic causes of mitochondrial disease with renal involvement.^12^ While this is true, clinical features are emerging that are suggestive of a particular aetiology—our case series shows that *RMND1* mutations are associated with both renal tubular acidosis type 4 (hyponatraemia and hyperkalaemia) and cystic/hypoplastic kidneys. In contrast, recessive mutations in *RRM2B* are associated with proximal tubulopathy (hyponatraemia and hypokalaemia),^13^ while steroid-resistant nephrotic syndrome (glomerular disease) is more commonly associated with primary coenzyme Q10 deficiency.^14^ Furthermore, normal mtDNA copy number in *RMND1* mutations is another important distinction compared with the nuclear genes that are responsible for mtDNA maintenance disorders such as *PEO1*, *RRM2B* and *TK2*. The clinical presentation of *RMND1* mutations may also mimic HUPRA syndrome (hyperuricaemia, pulmonary hypertension, renal failure in infancy and alkalosis) caused by mutations in *SARS2.*^15^ However, pulmonary hypertension was only identified in two of our patients, one had congenital heart defect (patent ductus arteriosus, P3) and the other developed pneumothorax at birth (P6) both of which would be risk factors for the development of pulmonary hypertension. In addition, metabolic acidosis was prevalent among those with renal disease and transient alkalosis was only identified in a patient (P1) who was negative for *SARS2* mutations.

*RMND1* mutations should also be considered as an important differential diagnosis to other inherited renal diseases, such as recessive Bartter syndrome type 4A (OMIM #602522) or dominant Familial Juvenile Hyperuricemic Nephropathy Type 2 caused by dominant mutations in *REN* (PMID: 19664745) (OMIM #613092). Although there are some overlapping clinical features, oligohydramnios, hyperkalaemia, arterial hypertension, mixed metabolic and lactic acidosis and significant neurodevelopmental delay are useful, discerning clinical pointers to *RMND1*-related mitochondrial disease.

In this study, we have identified six novel variants (c.533C>A, p.(Thr178Lys); c.565C>T, p.(Gln189*); c.631G>A, p.(Val211Met); c.1303C>T, p.(Leu435Phe); c.830+1G>A and c.1317+1G>T splicing variants) in the *RMND1* gene. The pathogenicity of these variants has highly likely given the following evidence: (1) the clinical phenotypes are compatible with the multisystem manifestation of mitochondrial disease, and associated with characteristic histochemical abnormalities and respiratory chain deficiencies in the muscle; (2) they affect highly conserved amino acids or are predicted to truncate the *RMND1*-encoded protein; (3) they are not common in a large number of ethnically matched control DNA samples (most variants are entirely novel, being absent on both ESP6500 and ExAC, with the exception of three rare variants—c.713A>G, p.(Asn238Ser) [21/120 626 alleles on ExAC and 5/12 982 alleles on ESP6500], c.1250G>A, p.(Arg417Gln) [1/119 954 alleles on ExAC, absent on ESP6500] and c.1349G>C, p.(*450Serext*31) [2/121 222 alleles on ExAC, absent on ESP6500]); (4) the parents of these patients are carriers of one variant and are clinically unaffected, thereby confirming segregation with disease, consistent with recessive inheritance.

Statistical analysis of genotype–phenotype correlations is limited by the small number of patients. We observe that the homozygous c.713A>G, p.(Asn238Ser) mutation cases (n=3) and compound heterozygous c.713A>G, p.(Asn238Ser) and c.1250G>A, p.(Arg417Gln) mutation cases (n=2) appear to have a more benign disease course (all are still alive, aged >6 years) compared with the other two groups; all patients harbouring either a homozygous c.1250G>A, p.(Arg417Gln) (n=4) or homozygous c.504+1G>A splicing variant (n=5) died within 12 months from birth. Disparity in clinical prognosis was most evident for those who harboured a homozygous c.1349G>C, p.(*450Serext*32) mutation, as three patients died within 12 months while the oldest patient survived beyond 6 years. This is difficult to explain in relation to *RMND1* protein expression, as this appears to be ubiquitous in all tissue types. Equally, why only certain variants such as c.1349G>C, p.(*450Serext*32) should be linked to bradycardia is also uncertain. Clearly, there are a wide range of clinical features associated with *RMND1* mutations, but the frequency of occurrence for each clinical feature varies enormously, with hypotonia, developmental delay and sensorineural hearing loss being the obvious exceptions.

In summary, the clinical phenotypes associated with *RMND1* mutations are more heterogeneous than that were initially described. We show that the congenital sensorineural deafness, central hypotonia, developmental delay and lactic acidaemia are cardinal clinical features associated with *RMND1* mutations. Regular monitoring of kidney function and blood pressure is imperative in the clinical practice in light of nephropathy being present in over 60% of cases. Furthermore, renal replacement therapy including kidney transplant should be considered particularly in those patients with mild neurological manifestation as shown in our study that four recipients of kidney transplant demonstrate good clinical outcome to date.

STROBE statement: STROBE guidelines were adhered to in the write-up and analysis of this observational, cohort study.
