# Supplementary material for: The clinical, biochemical and genetic features associated with RMND1-related mitochondrial disease
Source: J Med Genet. 2016 Jul 13;53(11):768–75. doi: 10.1136/jmedgenet-2016-103910 (PMC5264221; doi:10.1136/jmedgenet-2016-103910)
Supplement: Supplementary data [file jmedgenet-2016-103910supp.pdf]

Ng et al. The clinical, biochemical and genetic features associated with *RMND1*-related mitochondrial disease

Supplemental data 1

Patient 1: This female patient was the first child of non-consanguineous Italian parents and was born at 32 weeks gestation following a pregnancy complicated by severe oligohydramnios. A prenatal ultrasound scan revealed bilateral nephromegaly confirmed by another scan performed in the first day of life. Her weight at birth was 2,090 kg (90th centile for gestational age). The brainstem auditory evoked potential (BAEP) showed profound bilateral sensorineural deafness in the second week. She subsequently presented with recurrent episodes of vomiting and failure to thrive (weight <5th percentile).

She was noted to have normochromic normocytic anaemia with haemoglobin (Hb) of 64 g/L (normal value > 120 g/L), metabolic hypochloraemic hypomagnesaemic alkalosis, mild hyponatraemia (sodium level 130 mmol/L), renal impairment with mild proteinuria and hypertension aged 3 months. She received blood transfusion, fluid replacement therapy and antihypertensive drugs. Subsequent assessment and diagnostic work up at the metabolic unit revealed mild hypotonia, psychomotor delay and persistent lactic acidaemia further supported by hyperalaninemia and presence of lactic acid and Krebs'cycle intermediates in urine. Parathyroid hormone (PTH) levels were significantly increased (248 mg/dl, nv 14-72) with normal serum calcium level, suggestive of secondary hyperparathyroidism. Her creatinine kinase (CK), thyroid function test (TFT) and liver function test were normal. A renal ultrasound scan showed bilateral nephromegaly with microcystic and hyperechogenic appearance in the cortex and cortical-medullar junction as well as enlarged, globular liver, spleen and thyroid gland. Cranial MRI showed a picture of vacuolating leukoencephalopaty especially in the temporal-parietal areas. Visual evoked potentials and nerve conduction studies were unremarkable. Electrocardiography and echocardiography were normal.

Combined kidney, muscle and liver biopsies were performed at the age of 5 months. The key findings of renal biopsy were immature glomeruli (consistent with age), different degrees of mesangial sclerosis and various tubulo-interstitial changes, most notably ectasia, pseudo-cystic tubules and focal atrophy. Hyperplastic arteriolitis was also found in the interstitium. Muscle biopsy revealed COX-deficient muscle fibers and reduced complex IV enzymatic activity. There was no specific finding in the liver biopsy.

At one year old, she developed epileptic spasms with EEG finding of a pseudo-hypsarrhythmic pattern and responded well with low dose of vigabatrin and clonazepam. She also developed recurrent pancreatitis that required hospital admission for supportive treatment and serial abdominal ultrasound demonstrated enlarged and hyperechogenic pancreas. She had cochlear implant at 2 years old. On her last clinic visit (aged 3 years), she shows a clinical picture of moderate global psychomotor delay, microcephaly, sensorineural deafness, hypotonia, lower limbs hyperreflexia, failure to thrive (weight <3th centile) and dysphagia requiring percutaneous gastrostomy and chronic kidney disease (CKD). She is seizure–free and the antiepileptic treatment has been discontinued.

Targeted WES for mitochondrial disorders [1] revealed compound heterozygous mutations in the *RMND1* gene, a previously reported variant c.713A>G, p.(Asn238Ser) missense variant and a novel c.1303C>T, p.(Leu435Phe) variant.

Patient 2: He is the only child born to non-consanguineous Caucasian parents. The pregnancy was complicated by oligohydramnios and he was born at 37th gestational week. He has

developmental delay, hypotonia, bilateral sensorineural deafness, delayed visual maturation and lactic acidosis. Renal impairment was first noted at 1 year old and his renal ultrasound showed increased echogenicity in both kidneys. He required recurrent hospital admissions due to gastroenteritis at age 10 years. His kidney disease has advanced toward end-stage renal failure and he has also developed cardiomyopathy. He has been managed conservatively with fluid restriction and remained stable at present.

Patient 3: This female was born prematurely at 31 weeks gestation to non-related Irish parents. The pregnancy was complicated by oligohydramnios and poor foetal growth. She underwent emergency laparotomy and was found to have necrotizing enterocolitis at 14 days old. She was found to have pulmonary hypertension due to patent ductus arteriosus. At 5 months, she was admitted for diagnostic work up for persistent lactic acidosis, global developmental delay, microcephaly. Her cranial MRI showed delayed myelination and peak of lactate in MR spectroscopy. Hypertension, renal impairment, left ventricular hypertrophy and hearing impairment were also detected. He was commenced on PEG feeding due to failure to thrive. She had a muscle biopsy that showed complex I and IV deficiencies at 2 years old. She was not commenced on dialysis and listed for renal transplant in view of the multisystem involvement. She died following a presumed cardiac arrest at home at age 3.1 years.

Patient 4.1 is a boy born to non-consanguineous, Caucasian parents. His antenatal and birth history was unremarkable. He had feeding difficulty requiring multiple formula changes and was initially attributed to milk allergy in the early life. He was unable to hold his head up, roll over and crawl at 6 months and remained hypotonic at 16 months. It became clear he had difficulty with his hearing and audiology testing showed bilateral sensorineural hearing loss, and hearing aids were prescribed. The addition of speech delay and progressive lower limb spasticity led to the performance of cranial and spinal MRI head. The imaging showed leukoencephalopathy, temporal lobe cyst and possible lipomatous changes in the spinal cord. He developed first febrile seizure at aged 2.67 years and the EEG was not diagnostic. He had two further seizures which were also related to fever. He has since been prescribed clonazepam and has been seizure free. He is stable at 8 years old.

His younger brother (**Patient 4.2**) shared a similar clinical picture, however, also had torticollis and plagiocephaly required physiotherapy and regular botox injection. In addition, he showed a significant developmental regression with loss of ambulation and language at aged 4 years. He also developed febrile seizures and his EEG showed mild to moderately severe diffuse cortical dysfunction with temporal spike and waves. Levetiracetam was commenced with some observed improvement in the muscle tone and language skills but not sufficient to return him to his previous level. He is stable at age 6 years and has remained seizure free for 3 years. WES identified a homozygous c.713A>G, p.(Asn238Ser) variant in *RMND1* in Patient 4.1 and Patient 4.2.

Patient 5 is a girl born to non-consanguineous, mixed Caucasian and Native American parents. She was born prematurely at 35 weeks gestation and required admission to neonatal intensive care unit (NICU) due to hypoglycaemia, electrolyte abnormalities and recurrent apneas. She failed the hearing screen whilst on NICU and was subsequently diagnosed with bilateral hearing impairment for mid to high frequency range at one month old. Failure to thrive and gastro-oseophageal reflux were identified at the 2nd and 3rd month old respectively. Her CT head showed bilateral basal ganglia calcification with atrophy of the frontal and temporal lobes. These findings were initially thought to be consistent with Aicardi-Goutieres syndrome but genetic analysis for AGS1 (*TREX1*) and AGS2 (*RNASEH2B*) were negative.

At 4 months old she presented to the casualty department with projectile vomiting after feeds, decreased oral intake, failure to thrive and microcephaly (weight and head circumference were at 5th percentile). She was hypertensive (BP 150/100) and was only controlled with a combination of clonidine and isadipine. In addition, she had hyperkalaemia and hyponatraemia that were suggestive of renal tubular acidosis. A swallow study showed disorganized suck-swallow and no structural abnormality was identified in the oesophageal gastroscopy. She was commenced with gastrostomy feed. Serial cranial MRIs showed cerebral atrophy and white matter changes suggestive of delayed myelination but there was no brainstem abnormality.

She attended the paediatric neurology clinic for epilepsy, global developmental delay and central hypotonia, peripheral spasticity at 2.5 years. Clinical examination findings included alternating estropia, bilateral drooling but with normal facial strength, low central tone with increased tone peripherally and mild ankle contractures. There were possible focal dystonia affecting the right elbow and intermittent dystonic posturing of her fingers. There was no dysmetria or intention tremor but her grasp was clumsier with the left hand although the motor development was delayed on both sides. Primitive reflexes were absent but there was hyperreflexia and bilateral, non-sustained ankle clonus. She was unable to walk. She developed plagiocephaly likely as the result of a tendency lying on the right. Her sensorineural hearing deficit was progressive and was thought to be contributing to the speech and language delay. She had surgery for the strabismus around age four.

She was started on dialysis for end stage renal failure at aged 7 years. There were episodes of distress with hypertension, tachycardia, body flushing and low grade fevers suggestive of dysautonomia. Mild concentric left ventricular hypertrophy was detected. At this stage, her language skills remained very poor and she could only verbalize a few words but did respond to voice.

A homozygous c.713A>G, p.(Asn238Ser) mutation was identified through exome sequencing at the age of 8.5 years. She underwent renal transplant at 8.75 years old and her overall health and quality of life have improved considerably.

Patient 6 is born to non-related parents (Caucasian and Mexican). Shortly after birth, he developed respiratory distress, was found to have a pneumothorax and pulmonary hypertension. He required intubation and extracorporeal membrane oxygenation (ECMO) for 9 days and ventilator support for 17 days. At 4 months, he was diagnosed with hypotonia and motor delay. At 9 months, he was diagnosed with sensorineural hearing loss. Cochlear implants were placed at 13 and 15 months. He had a cranial MRI prior to cochlear implant which was interpreted as delayed myelination as well as "hypoxic ischemic encephalopathy" from birth injury. At 14 months, he underwent surgery to correct strabismus.

At 18 months, he was diagnosed with failure to thrive for poor weight gain, refractory gastro-oesophageal reflux and feeding aversion. A gastrostomy tube was placed. Further evaluation revealed evidence of renal tubular acidosis (hypertension, hyperkalemia, hyponatremia, hypoaldosteronism) and mild lactic acidosis (1.7-5.5 mMol/L).

At 29 months, due to global developmental delays associated with hyperreflexia, he had a muscle biopsy. Histology showed only mild focal type II fiber hypotrophy and no COX-fibers. His mtDNA copy number was markedly increased to 239% of control as well as citrate synthase level (258% of control) Biochemical studies showed complex IV 10% mean after correcting for CS; Complex I 52%, I+III 50%; II+III 46%. Fibroblast studies were unremarkable.

He progressed to chronic kidney disease. By age 4, he had developed hypertrophic cardiomyopathy and it was unclear whether this was secondary to persistent hypertension or underlying mitochondrial disease. At age 4.5 years, he developed suspected haemolytic uraemic syndrome following hyperbaric oxygen therapy which was responsive to eculizimab. His chronic kidney disease progressed to end stage renal failure requiring dialysis by age 5 years and 4 month. His renal biopsy showed changes consistent with chronic tubulointerstitial nephropathy with tubular atrophy (50%), interstitial fibrosis, mild chronic inflammation, and focal proximal tubular vacuolation with secondary focal global glomerulosclerosis.

At age 5, he was able to ambulate with assistance (walker); then he had a motor regression with H1N1; could no longer scoot age 6; cardiomyopathy progressed during H1N1 illness at age 6 (ejection fraction fell from 50% to 20%). He underwent cardiac catheterization as part of the work up for renal transplant at age 6 years and two months. His heart biopsy showed marked cardiomyocyte hypertrophy without fibrosis and interestingly the electron microscopy showed normal architecture of mitochondria. He received a kidney transplant at age 6 years 5 months. He has had much motor improvement after renal transplant. He is behind in school but trying to attend 1st grade with an individualized education plan. He remains well at 8 years.

Other symptoms include constipation managed with laxative (polyethylene glycol 3350), myopia corrected with glasses, microcephaly and low bone density on DEXA.

WES revealed a previously reported missense mutation c.713A>G, p.(Asn238Ser) and a c.1317+1G>T splice mutation in *RMND1*.

Patient 7.1 is a girl born to non-related Spanish parents. The initial pregnancy was complicated by threatened abortion, but she was born at full term with normal weight (3.03kg). Motor delay was detected in the first year of life with subsequent diagnosis of mild global neurodevelopmental delay including speech and language at 3 years old. Cranial MRI showed signal changes in the deep and periventricular white matter, together with subcortical hyperintensities in anterior temporal white matter which was more pronounced in the right temporal lobe. Bilateral sensorineural hearing loss was detected and she was prescribed hearing aids around the same time. Blood tests revealed anaemia (Hb 96g/L), hyperuricaemia (7 mg/dl; normal values 3.71±0.92 for female 5-9 years old) and mild hyperkalaemia. A metabolic study of purine metabolism was performed without any abnormal finding in purine and pyrimidine urinary excretion. There was a hypopigmented lesion detected in her back.

She developed stage 2 CKD and was referred to a paediatric nephrologist at 7 years old. Her estimated glomerular filtration rate (eGFR) was 80 ml/min/1.73m^2 without evidence of proteinuria. She had persistent mild hyperkalemia (6 mmol/; normal values 3.5-5) and hyperuricaemia high blood uric acid levels 9.8 mg/dl, with a low fractional excretion 5.4% (normal values 10±3 for female 5-9 years old). Metabolic acidosis (pH 7.24, bicarbonate level 19 mmol/L) with normal anion gap was evident and plasma lactate was normal. Urinary pH was 5. Renal ultrasound scan showed asymmetry of renal size suggestive of left renal hypoplasia and normal echogenicity. Her urine concentrating capacity was nearly normal achieving maximal osmolality value of 778 mosm/kg in first morning void. Hyperuricaemic nephropathy, familial juvenile 2 (HNFJ2) was suspected, but she tested negative for mutations in the *REN* gene.

She receives conservative management with allopurinol, sodium bicarbonate, vitamin D, iron and darbepoetin but her eGFR has declined gradually to 66 ml/min/1,73m^2 at age of 11 years.

She has no neurological deficit and neuropsychological assessment showed IQ of 80 at aged 10. Growth hormone therapy has been commenced for short stature (height 121 cm, 2nd centile) since age 10 and her height has improved to 25th centile.

Patient 7.2 is the younger sister of patient 7.1. She also presented with developmental delay at 18 months and cranial MRI findings were similar to her sister at age 4. Sensorineural hearing loss was initially suspected but was only confirmed at 3 years old. It progressed faster than her sister and she received cochlear implants at 6 years old. She had mottled hypopigmented lesions in her left thigh suggesting blascholinear dermatosis and a "blonde dyed-like frizzy hair" in which microscopy exam revealed pili torti, which was also confirmed in her sister, whose hair appeared macroscopically normal.

Renal involvement was first noted at 4 years old. Her eGFR was normal but there were hyperuricaemia, mild hyperkalaemia, anaemia (Hb 101 g/L) and metabolic acidosis without raised plasma lactate. She has received similar management to her sister, however, her CKD appears to have progressed faster to stage 3 at 8 years old. Abdominal ultrasound scan showed hyperechogenic pancreas and cortical hyperechogenity in the right kidney. Her height and growth are normal. There is no neurological problem.

WES identified compound heterozygous *RMND1* mutations, c.713A>G, p.(Asn238Ser) and c.1250G>A, p.(Arg417Gln) in both sisters.

Patient 8: This girl is the only child of healthy, non-consanguineous parents. She was born by a normal delivery at term with a birth weight of 2.66 kg. She presented at 13 hrs of age with hypoglycaemia (1.6 mmol/L) and lactic acidaemia (14 mmol/L, rising to 29 mmol/L with a lactate:pyruvate ratio of 40). The ammonia concentration was 84 μmol/L, the urine organic acid, plasma amino acid and blood acylcarnitine profiles were unremarkable apart from a low free carnitine, which subsequently returned to normal. Echocardiography and cranial ultrasound examination were normal. The initial hypoglycaemia and acidosis were corrected and the lactate concentration dropped to 4 mmol/L, though it rose again during minor illnesses.

Initially, the patient fed orally but from 9 months of age she has been tube fed through a gastrostomy. Newborn screening revealed severe sensorineural hearing loss. She has abnormal visual behaviour but briefly follows a bright light with her eyes; no ophthalmological abnormalities were found and the impaired vision is thought to be of cerebral origin. She has developed a variable divergent squint. From 11 months of age, she has had seizures that resemble infantile spasms. The EEG showed lots of spike and wave but not hypsarrhythmia. She has been treated with levetiracetam which has greatly reduced the seizures. The head circumference was normal at birth but she developed severe microcephaly. Muscle tone is normal but she has increased reflexes in the legs. Cranial MRI aged 6 months showed no structural or focal white matter abnormality. The ventricular system was of normal size & configuration but the extra-axial CSF spaces around both cerebral hemispheres appeared prominent.

Sodium and magnesium supplements were required from the neonatal period but it was possible to stop the magnesium supplements at 3 years of age. She developed chronic renal impairment and hypertension, which required treatment with amlodipine until 3 years of age, when this was withdrawn without recurrence of the hypertension. Ultrasound showed normal sized kidneys with no focal scars but increased echogenicity. At the age of 3 years 9 months, her clinical state was remarkably stable. Her urea was 22.4 mmol/L and creatinine 174

μmol/L. Cystatin C was elevated to 3.80mg/L. Her estimated GFR is lower than 18ml/minute per 1.73m^2 (CKD stage 4). Urate was increased to 436 μmol/L and PTH was elevated to 13.0 pmol/L despite treatment with alphacalcidol.

Respiratory chain studies in muscle showed markedly reduced activity of Complex IV (0.6 x10^{-3} K/s/unit citrate synthase, normal1.12±0.51); activity of the other complexes was normal. Muscle histochemistry showed absent cytochrome c oxidase activity in all fibres. Cytochrome c oxidase activity was also low in fibroblasts.

Patient 9: The proband was 4 year old boy who initially presented with hypotonia, hearing loss and seizures. He was born at term to consanguineous healthy parents (first cousins) after an uneventful pregnancy. Two of his siblings had suffered from the same condition and died due to the disease complications (genetically undetermined). The patient had irregular heartbeat. Holter monitoring revealed sinus rhythm with junctional escape (first degree block). Echocardiography showed dilated globular left ventricle with preserved LV function (LVEF: 62.7%). His neurodevelopmental status was normal. Kidney ultrasound revealed bilateral hyperechoic kidneys with calcification in renal parenchyma and very poor corticomedullary differentiation. Biochemical tests rereleased lactic acidosis and electrolyte imbalance (hyperkalemia: 8.3 mmol/L (normal range 3.5-5.1 mmol/L), low carbonate level 11 mmol/L (normal range 22-32 mmol/L), high creatinine:2 mg/dL (normal range 0.3-0.7 mg/dL) and high blood urea nitrogen: 79 mg/dL (normal range: 6-20 mg/dL).Whole exome sequencing on the DNA samples of the patient identified a G to C mutation (p.X450SerextX32) in *RMND1* gene that results in a stop-loss and adds 32 amino acids to the protein.

Patient 10.1 was the second child born to consanguineous Bangladeshi parents. The pregnancy and delivery were uneventful. Sensorineural hearing loss was first detected via screening. Motor developmental delay was commented at 5 month old. At 8 months, she presented with acute gastroenteritis with hypotonia, profound metabolic acidosis, hyperkalaemia (highest potassium level was 8mmol/L), renal failure and heart block. She was anaemic with a haemoglobin of 70g/L and her creatinine kinase level was mildly raised at 568 IU. A permanent pacemaker was inserted for the bradycardia and pericardiocentensis was performed to relieve the pericardial effusion. She had an epicardial biopsy and hypertension was identified during the same admission. She also required nasogastric feed due to dysphagia. Nerve conduction studies and electromyopraphy excluded spinal muscular atrophy and congenital myaesthenic syndrome. MRI head was suggestive of some degrees of brain volume loss given prominent ventricles and increased extra-axial fluid spaces. However, no other abnormality such as white matter changes or basal ganglia abnormality was identified.

She died from multi-organ failure precipitated by sepsis just below one year old.

Patient 10.2: She was the older sister of patient 10.1 who initially presented at the age of 9 months with vomiting and irritability. Like her sister the pregnancy and delivery were uneventful but postnatal growth had been poor. She had gross motor delay and was not sitting without support at this age. During this illness she had a marked hyperchloraemic metabolic acidosis, bradycardia and hypertension requiring labetalol infusion initially. Even before antihypertensive treatment was started, she had persistent bradycardia and electrocardiography revealed variable degrees of heart block. Bilateral profound sensorineural hearing loss had been demonstrated and an MR brain imaging suggested delayed myelination. A mitochondrial disorder had been suspected given the multisystem

problems at presentation but further investigations had not been performed prior to the family's transfer to our unit at the age of 16 months when she had a further acute admission with vomiting, hypertension and acidosis. At this point her lactate was higher, ranging from 3.6-7.1 mmol/L (normal range 0.6-2.2 mmol/L) and she showed evidence of chronic renal failure. She had a pacemaker insertion and renal biopsy. Urine organic acids showed persistent excretion of 2-oxoglutarate suggestive of a disorder of mitochondrial function. Over the next 2 years, she showed steadily decreasing renal function with particular evidence of renal tubular acidosis (sodium and chloride loss requiring supplementation – lowest Na 108 mmol/L, lowest Chloride 52 mmol/L and mild hyperkalaemia max 5.9 mmol/L) and her peak creatinine rose to 410uM. She demonstrated marked renal osteodystrophy (serum parathyroid hormone levels >2500 ng/L [normal range 11-35]) which responded partially to supplementation with 1-alpha-hydroxycholecaliferol. She demonstrated a persistent normo- to macrocytic anaemia with a minimum Haemoglobin of 49 g/L prior to transfusion (normal range 11.7-13.7). Given her multisystem disorder, active management of end stage renal failure was not instituted and the child received palliative care, passing away at the age of three years without a genetic diagnosis. She was also found to harbour the homozygous c.1349G>C, (p.*450Serext*31) mutation after the same mutations were identified subsequently in her younger sister through WES.

Patient 11.1: This girl was the third child born to healthy, related Pakistani parents. Her mother had had one early pregnancy termination for multiple congenital anomalies. The pregnancy was uneventful. She was born at 37 weeks gestation with a birth weight of 3.06 kg. She failed the neonatal hearing test and was diagnosed with bilateral profound sensorineural hearing loss at one month of age. At 8-month old, there were concerns regarding developmental delay as she was unable to roll over, sit independently or crawl and was noted to be hypotonic. She became less interactive and stopped smiling afterwards. Her weight was on the 9th percentile and her head circumference was on the 25th percentile aged 11 months. She had developed moderate dysphagia and required nasogastric feeds. She developed bradycardia due to first and second degree heart block. She had persistent lactic acidosis (7.0-9.9 mmol/L). Her urine metabolic screen and normal urine amino and organic acids were normal. Plasma carnitine was normal but several acylcarnitines were increased, including acetyl and hydroxybutyryl carnitines, consistent with ketosis or lactic acidosis. There were peaks in the position of the dicarboxyl acyl carnitines possibly due to renal dysfunction. Her TORCH screen was normal. Cranial MRI including spectroscopy was normal at aged 11 months.

She died following a presumed cardiac arrhythmia at the age of 16 months. Post mortem examination noted she was microcephalic with a head circumference below the 3rd percentile. She had cardiomegaly with irregular muscle fibres on histology, acute focal bronchopneumonia of left lower and right upper lobes of the lung. There was an acute on chronic stress reaction of the thymus and cystic changes in the kidney cortex.

She was found to have a homozygous c.1349G>C, (p.*450Serext*31) in *RMND1* on whole exome sequencing. She had a cousin (P11.2 in Table 1) who also harboured the same mutations as reported previously [2].

Patient 11.2: This female child was the older cousin of patient 11.1 and was born to consanguineous Pakistani parents at 37 weeks gestation with normal weight (3.47kg) and head circumference (50th centile). Sensorineural deafness was detected and hearing aid fitted by one month old. She presented to the paediatric team with hypotonia, failure to thrive and

poor head growth aged 3 months, with initial investigations showing hyponatraemia, hyperkalaemia, anaemia and raised serum lactate 3.5 mmol/L. This prompted planned inpatient admission for further assessment. Clinical examination (aged 4 months) revealed weight of 4.7kg (0.4 centile), microcephaly (head circumference of 39.3 cm, 2nd centile) and hypotonia but she was alert, able to track and smile. Laboratory investigations showed anaemia (72g/L), hyperkalaemia (up to 7.8mmol/L), low bicarbonate (10mmol/L, normal range 20-28 mmol/L), urea 4.8, creatinine 48 μmol/L, marginally raised CSF lactate at 2.5 mmol/L (paired serum lactate 3.5 mmol/L) and CK level 216 IU. Short synacthen test was normal. Blood film showed red cell fragments, acanthocytes and burr cells whilst bone marrow biopsy showed changes suggestive of a secondary anaemia. Her renal ultrasound was unremarkable. Muscle histochemistry showed severe COX deficiency with biochemically-confirmed combined complex I and IV deficiencies.

At aged 7.5 months, she was commenced on nasogastric feed due to failure to thrive. The microcephaly persisted with head circumference below the 0.4th centile. She was extremely hypotonic (trunk and limbs). There was no report of seizures.

Her weight was still 4.9kg by one year old. She died from respiratory failure shortly afterwards. This patient had an older brother who had seizures, was ventilated & died aged 17 days but who did not have the *RMND1* mutation. There is also a healthy brother & sister.

Patient 12: This girl was born to related Pakistani parents. She was admitted under the care of paediatric team due to failure to thrive and malnutrition aged 20 months. Clinical assessment revealed global developmental delay, microcephaly, sensorineural deafness, hypotonia, myopathic facies and areflexia. She developed heart failure due to cardiomyopathy that required intravenous inotropic support for several months but this stabilized by 2 years of age. Further investigations showed renal tubulopathy with proteinuria (urine protein:creatinine ratio 1838) and cystic dysplasia was identified on the abdominal ultrasound. She developed acute right hemiplegia and her MRI head showed left thalamostriate infarct at 23 months of age. Her blood lactate level was normal. Her muscle biopsy showed fibrosis and necrosis, marked COX deficient fibers and profound multiple respiratory chain deficiencies.

She made a fairly good recovery from her hemiplegia: at 6.5 years, she could walk short distances & communicate by sign language. She died suddenly aged 6 years 8 months.

Patient 13 was born to consanguineous, healthy Pakistani parents. The pregnancy was uneventful and her birth weight was 3kg. Problems with feeding, hypotonia and failure to thrive were noted several months later. She was admitted to hospital for assessment which showed generalized hypotonia, axial weakness, sensorineural hearing loss and normal eye movement at 6 months old. She was interactive and able to smile. This admission was complicated by aspiration pneumonia and she was commenced on nasogastrostomy feed as her weight was only 6.8kg. She was hypertensive and bradycardic with serial ECGs showing 2nd to 3rd degree heart block. Further investigations revealed hyponatraemia, hyperkalaemia (4.1 to 8.3 mmol/L), raised creatinine kinase (900-1507 IU), lactic acidaemia (3.74 to 11.1 mmol/L), normal CSF lactate (1.4 mmol/L), normal serum acylcarnitine level, normal random cortisol level and hormonal profiles. Her renal ultrasound showed abnormal echogenicity suggestive of underlying parenchymal disease. MRI head was reported as normal. She underwent a muscle biopsy which showed lipid vacuoles and multiple respiratory chain deficiency, highly suggestive of a mitochondrial disorder.

She subsequently developed diarrhoea, worsening lactic acidosis and respiratory failure despite supportive treatment on the ward. Her arterial blood gas showed profound, mixed respiratory and metabolic acidosis (pH of 7.02, pCO_2 14.1kPa, HCO_3 19.3 mmol/L, base excess -5.1 and lactate 18.5 mmol/L). In view of multisystem involvement, escalation of care was deemed inappropriate and she died very shortly after.

Patient 14: This was a 5th child of the consanguineous Pakistani parents, who was born with normal delivery. He failed the neonatal hearing test at birth. There was a concern of motor developmental by 6 months as he was unable to sit though that improved by 10 months. At thirteen months old, his occipital-frontal circumference (OFC) was charted at 9-25th centile and weight was at 0.4-2nd centile. He was still unable to fully support his body weight, just manageing to crawl with high kneeling by 2 years. Percutaneous endoscopic gastrostomy feed was commenced for failure to thrive at age 4. He had raised serum lactate (ranged from 3.51 to 6.97 mmol/L) but with normal CSF lactate levels (2 and 1.9 mmol/L on two occasions). He also had hyperkalaemia (highest level was 6.3 mmol/L) and raised CK level (463-1125 IU). Dysplastic kidneys with abnormal echogenicity were identified on abdominal ultrasound.

He became hypertensive and developed heart block, tricuspid regurgitation and right sided heart failure. He underwent a muscle biopsy which showed subsarcolemmal accumulation of mitochondria with abnormal appearance and crystal formation under the electron miscopy study. Multiple respiratory chain deficiencies was also detected.

His kidney disease progressed to end stage renal failure at age 5. He was not considered for kidney transplantation due to the pre-existent heart block and heart failure. He died from pneumonia and multi-organ failure at 5.8 years.

Patient 15: A six month old female child of consanguineous Pakistani parents presented with myopathic facies, tented upper lip, sagging cheeks, weak cry, hypotonia (axial more marked than appendicular), and generalized muscle weakness (MRC grade 3/5). Deep tendon reflexes were present. There was a history of failure to thrive, developmental delay, congenital bilateral sensorineural hearing loss, and bilateral cystic dysplastic kidneys. She subsequently developed junctional rhythm with nocturnal bradycardia. There were no eye abnormalities. Blood investigations showed persistently high urea up to 29mmol/l (range 2.5-6.5), raised lactate (8.3mmol/l; range 0.6-2.5), pyruvate (232 μmol/l; range 41-114), and lactate:pyruvate ratio (28.8; normal range <25). Cerebrospinal fluid protein and lactate were normal. MRI brain at age 6 months was normal. Muscle biopsy examination showed features of mitochondrial disorder with evidence of complex I, III and IV deficiencies. On molecular genetic analysis for mitochondrial respiratory chain defects, she was found to be homozygous for a novel c.1349G>C, (p.*450Serext*31)in *RMND1*. She died aged 2 years following respiratory failure associated with respiratory tract infection.

Patient 16: This female child was born at 41+3 weeks with normal weight (3.45kg). She had neonatal jaundice, was a poor feeder and described as an irritable unsettled baby. At 6 weeks, she was diagnosed with profound, bilateral sensorineural deafness, alternating squint and developmental dysplasia of hip (DDH) which was treated with hip spica under general anaesthesia (GA). She made a slow recovery from GA and developed acute hyponatraemia & hyperkalaemia in the post-operative period which was treated with sodium supplements. She later was given a trial of chlorthiazide and sodium bicarbonate which proved successful.

By 6 months of age there were concerns about her development. MRI head showed cystic changes in the right temporal lobe and periventricular white matter changes, suggestive of poor myelination throughout. She was subsequently referred to neurology due to significant, global developmental delay, only had pre-verbal communication with parents and microcephaly. She was found to have spastic diplegia (lower limbs).

She was formally referred to nephrology team with recurrence of hyponatraemia identified at the pre-op assessment for insertion of cochlear implant at 20 months. She developed acute tubular necrosis following an acute vomiting illness. She had a renal biopsy that showed changes that were consistent with interstitial nephritis. Her kidney disease progressed to stage 4 and the hyponatraemia, hyperkalaemia and hypertension were controlled by medication at age 5. She did not receive renal replacement therapy. She was commenced on gastrostomy feeding at age 5.

She deteriorated acutely with an inter-current viral gastroenteritis. She developed acute liver failure, with worsening renal function. She became encephalopathic with refractory acidosis. She died peacefully with her parents at age 5.5.

References:

1. Lieber DS, Calvo SE, Shanahan K, Slate NG, Liu S, Hershman SG, Gold NB, Chapman BA, Thorburn DR, Berry GT, Schmahmann JD, Borowsky ML, Mueller DM, Sims KB, Mootha VK. Targeted exome sequencing of suspected mitochondrial disorders. Neurology 2013;**80**(19):1762-70 doi: 10.1212/WNL.0b013e3182918c40.
2. Taylor RW, Pyle A, Griffin H, Blakely EL, Duff J, He L, Smertenko T, Alston CL, Neeve VC, Best A, Yarham JW, Kirschner J, Schara U, Talim B, Topaloglu H, Baric I, Holinski-Feder E, Abicht A, Czermin B, Kleinle S, Morris AA, Vassallo G, Gorman GS, Ramesh V, Turnbull DM, Santibanez-Koref M, McFarland R, Horvath R, Chinnery PF. Use of whole-exome sequencing to determine the genetic basis of multiple mitochondrial respiratory chain complex deficiencies. JAMA 2014;**312**(1):68-77 doi: 10.1001/jama.2014.7184.
